# Modifying Thermostability and Reusability of Hyperthermophilic Mannanase by Immobilization on Glutaraldehyde Cross-Linked Chitosan Beads

**DOI:** 10.3390/biom12070999

**Published:** 2022-07-18

**Authors:** Beenish Sadaqat, Chong Sha, Mudasir Ahmad Dar, Maruti J. Dhanavade, Kailas D. Sonawane, Hassan Mohamed, Weilan Shao, Yuanda Song

**Affiliations:** 1 Colin Ratledge Center for Microbial Lipids, School of Agriculture Engineering and Food Science, Shandong University of Technology, Zibo 255049, China; beenish@sdut.edu.cn (B.S.); hassanmohamed85@azhar.edu.eg (H.M.); 2 School of the Environment and Safety Engineering, Biofuels Institute, Jiangsu University, Zhenjiang 212013, China; jnshachong@163.com (C.S.); mudasir.dar@unipune.ac.in (M.A.D.); 3 Department of Microbiology, Bharati Vidyapeeth’s Dr Patangrao Kadam Mahavidyalaya College, Sangli 416416, India; marutijd@gmail.com; 4 Structural Bioinformatics Unit, Department of Biochemistry, Shivaji University, Kolhapur 416004, India; kds_biochem@unishvaji.ac.in; 5 Department of Botany and Microbiology, Faculty of Science, Al-Azhar University, Assiut 71524, Egypt

**Keywords:** β-mannanase, docking, hyperthermostable, kinetics

## Abstract

In the current study, the purified β-mannanase (Man/Cel5B) from *Thermotoga maritima* was immobilized on glutaraldehyde cross-linked chitosan beads. The immobilization of Man/Cel5B on chitosan beads was confirmed by Fourier-transform infrared spectroscopy (FTIR) and X-ray diffraction (XRD) analysis. After immobilization, the protein loading efficiency and immobilization yield were found to be 73.3% and 71.8%, respectively. The optimum pH for both free and immobilized enzymes was found to be pH 5.5. However, the optimum temperature of immobilized Man/Cel5B increased by 10 °C, from 85 °C (free Man/Cel5B) to 95 °C (Immobilized). The half-life of free and immobilized enzymes was found to be 7 h and 9 h, respectively, at 85 °C owing to the higher thermostability of immobilized Man/Cel5B. The increase in thermostability was also demonstrated by an increase in the energy of deactivation (209 kJmol^−1^) for immobilized enzyme compared to its native form (92 kJmol^−1^), at 85 °C. Furthermore, the immobilized Man/Cel5B displayed good operational stability as it retained 54% of its original activity after 15 repeated catalytic reactions concerning its free form.

## 1. Introduction

Mannans are an integral part of hemicellulose and are predominantly found in the soft woods, plant endosperms, seeds, and vacuoles of a wide variety of plants [1,2,3]. Mannans are also present as glycoproteins in the cell walls of some yeasts, fungi, and bacteria [4]. β-1,4-mannanase (mannan mannohydrolase, EC 3.2.1.78) is an endo-acting hydrolase that randomly cleaves the β-1,4-mannosidic linkages in the main chain of mannans (linear mannan, galactomannan, glucomannan, and galactoglucomannan), therefore producing mannooligosaccharides (MOS) of various lengths [5]. β-mannanase has wide industrial applications as it is used for the production of partially hydrolyzed guar gum, MOS, fruit juice clarification, paper/pulp bio-bleaching, synthesis of detergents, amelioration of animal or poultry feed, and saccharification of biomass [6,7].

Despite the versatility of β-mannanase, the use of these enzymes in soluble form at the industrial scale has some limitations including low stability, unmanageable recovery and reuse, short shelf life, difficulty in handling, and loss of activity at prolonged operational conditions [8]. However, immobilization of enzymes on or into solid matrixes can address these issues. It should be noted that immobilization may not always bring desirable changes to the enzyme system. The immobilization protocol must consider several aspects, such as properties of support material, the active group of the support used to immobilize the enzyme and that of the enzyme molecule, and the immobilization protocol used. Only a proper selection of these three aspects allows one to take full advantage of the immobilization process [9].

Immobilization can enhance the thermostability, pH stability, functional stability, and affinity of these biocatalysts [10]. In addition to this, immobilization enables easy recovery of biocatalysts from the end product and provides opportunities for scaling up and enabling the development of processes based on different reactor configurations and reduction of operating costs [11]. Previously, β-mannanases have been immobilized on different support materials which include sodium alginate-grafted β-cyclodextrin [12], chitosan magnetic nanocomposites [13], cross-linked enzyme aggregates [14], and calcium alginate beads [15].

The efficiency of immobilization largely depends on the interaction of enzyme and support matrix. Therefore, the type of immobilization matrix plays a very important role in deciding the characteristics of the resultant immobilized biocatalysts [16]. The immobilization process may be of a physical nature, which includes adsorption, entrapment, and encapsulation of enzyme on or inside support matrix [17] or it may be of chemical nature which involves crosslinking and covalent bonding [18]. Numerous inert materials could be used for effective immobilization of enzymes because of their ability to reuse, easy recovery, higher thermal stability, and ability to survive under varying pH conditions [19]. Chitosan is one of the most widely used support materials for enzyme immobilization because of unique properties such as the presence of a large number of functional groups, ease of availability, biodegradability, and chemical resistance [20]. Chitosan beads have been widely used for the immobilization of β-mannanase which resulted in enhanced activity, stability, and reusability of enzyme. The presence of amino groups in chitosan structure also allows modification by crosslinking process. Glutaraldehyde is the most frequently used crosslinking agent which can further enhance rigidity, thermal stability, and absorptivity of chitosan support material. The covalent immobilization of enzyme on chitosan occurs by treating it will glutaraldehyde crosslinker. The bifunctional –CHO groups of glutaraldehyde react with the –NH2 groups present in chitosan and in the enzyme [21]. Işık et al. [22] used glutaraldehyde cross-linked chitosan beads for covalent immobilization of acetylcholinesterase (AChE) enzyme which resulted in high stability and reusability of enzyme.

In the current study, hyperthermostable β-mannanase from *T. maritima* (Man/Cel5B); which was purified and characterized in a previous study [6]; was immobilized on glutaraldehyde cross-linked chitosan beads. Properties of the immobilized and free Man/Cel5B were compared. Moreover, the kinetic and thermodynamic parameters of both forms of the Man/Cel5B were determined by applying different statistical tools to predict and compare their behavior under different environmental conditions. Finally, the interactions between ligand and the Man/Cel5B were studied using in silico structure-based molecular docking methods.

## 2. Materials and Methods

### 2.1. Chemicals

Chitosan (average molar weight, 4.9 × 105, degree of deacetylation 95%), locust bean gum (LBG), and glutaraldehyde (≥98% purity) were purchased from Sigma Chemical Co. (St. Louis, MO, USA). All other chemicals were of analytical grade and were purchased from Sinopharm Chemical Reagent Co. Ltd. (Shanghai, China).

### 2.2. Chitosan Beads Preparation and Immobilization of Man/Cel5B

Chitosan beads were prepared according to the procedure described by Kaushal [23] with slight modifications. Briefly, 2.5 g of chitosan was dissolved in 100 mL of 1% (*v*/*v*) acetic acid solution using continuous stirring till the solution was clear from any solids. The chitosan solution was added dropwise into 2% (*w*/*v*) of NaOH solution and beads were collected from precipitating solution. The prepared beads were placed in 100 mL of crosslinking solution for almost 3 h at 25 °C. The crosslinking solution was prepared by adding 0.2 vol.% of glutaraldehyde to 100 mL of the ddH2O. The crosslinked beads were filtered and stored at 4 °C for future use. The beads were lyophilized and surface morphology was observed using scanning electron microscopy on a SEM, Quanta FEG 250 machine [24].

For immobilization of Man/Cel5B onto chitosan beads, 1 mL of Man/Cel5B (purified near homogeneity in previous study) [6] was mixed with 9 mL of PI buffer (pH 6.8) and added to approximately 15 g of crosslinked chitosan beads previously stored at 4 °C. The cross-linked chitosan beads were placed in an enzyme solution (0.364 mg/mL) for 5 h at 30 °C in a rotary shaking incubator. The beads were then washed with cold phthalate-imidazole (PI; pH 6.8) buffer and stored at 4 °C for future use.

### 2.3. Activity of Immobilized Man/Cel5B Enzyme

The activity of immobilized Man/Cel5B was determined via 4-hydroxybenzoic acid hydrazide method [25]. The reaction mixture, containing 0.2 g of immobilized Man/Cel5B chitosan beads, 100 µL of 0.5% (*m*/*v*) LBG as substrate, and 100 µL of 0.25 M PI buffer (pH 5.5); was incubated for 5 min at the target temperature (e.g., 60–95 °C for evaluation of optimum temperature). The beads were removed, and 600 µL of stop solution was immediately added to stop the reaction. For free enzyme, 100 µL of Man/Cel5B was added to the reaction mixture instead of 0.2 g of beads. The mixture was boiled for 10 min and the absorbance was measured at 405 nm after cooling the reaction mixture on ice (0 °C). One unit of enzyme is the amount that produced 1 µmol of mannose sugar per minute under the said assay conditions [21].

### 2.4. Estimation of Protein Loading and Immobilization Yield

The total protein concentration was determined by measuring the absorbance at 280 nm [26]. The total amount of protein that immobilized on chitosan beads was estimated by subtracting the total protein concentration of supernatant (after immobilization of beads) from the protein concentration of free enzyme. In this regard, protein loading efficiency (%) and immobilization yield (%) were calculated by the following equations:

(1) $$\mathrm{Protein}\;\mathrm{Loading}\;\mathrm{efficiency}\;\left(\%\right)=\frac{\mathrm{Total}\;\mathrm{amount}\;\mathrm{of}\;\mathrm{immobilized}\;\mathrm{enzyme}}{\mathrm{Total}\;\mathrm{amount}\;\mathrm{of}\;\mathrm{initial}\;\mathrm{enzyme}}\times 100$$

(2) $$\mathrm{Immoblized}\;\mathrm{yield}\;\left(\%\right)=\frac{\mathrm{Specific}\;\mathrm{activity}\;\mathrm{of}\;\mathrm{immoblized}\;\mathrm{enzyme}}{\mathrm{Specific}\;\mathrm{activity}\;\mathrm{of}\;\mathrm{free}\;\mathrm{enzyme}}\times 100$$

### 2.5. Biochemical Characterization of Immobilized Man/Cel5B

#### 2.5.1. Effect of Temperature on Immobilized Man/Cel5B Activity

The impact of temperature on immobilized enzyme was verified by determining the enzyme activity at a temperature ranging from 60 to 100 °C with a temperature difference of 5 °C. The reaction set showing the highest enzyme activity was considered as 100% activity and the relative activity was determined concerning the maximum enzyme activity. The activation energy (E_act_) was evaluated from the slop of Arrhenius plot (ln of % relative activity versus 1000/absolute temperature) using the following equation:

(3) $$E_{\mathrm{act}}=-\mathrm{slope}\;\times \;R$$

where R is the gas constant (8.314 J·K^−1^ mol^−1^), and E_act_ represents activation energy (kJ·mol^−1^). The impact of temperature on free enzyme was also determined using same conditions and compared with the immobilized enzyme

#### 2.5.2. Effect of pH on Enzyme Activity

The optimum pH for both free and immobilized enzyme was examined at pH ranging from pH 4.0 to 7.5, using 25 mM PI buffer at the optimal temperature. The reaction set showing the highest enzyme activity was taken as 100% activity, and the relative activity was determined concerning the maximum enzyme activity.

#### 2.5.3. Kinetics and Thermodynamics of Substrate Hydrolysis

The kinetic parameters of free and immobilized enzymes were calculated by observing the activity in increasing substrate concentration i.e., 0.5–15.0 mg/mL. The activity was evaluated at the optimum temperature and pH. The Michaelis–Menten constant (K_m_) and maximum rate of reaction (V_max_) were determined using the Michaelis–Menten (V vs. [S]) and Lineweaver-Burk plots (1/*V* vs. 1/[S]). The turnover number (k_cat_) was calculated by the equation given below:

(4) $$k_{\mathrm{cat}}=V_{\max}/\left[E_{t}\right]$$

where Et is the total enzyme concentration.

The thermodynamic parameters, change in enthalpy (∆H), change in entropy (∆S), and Gibb’s free energy (∆G) were used to understand the behavior of enzymatic reactions and interactions and were calculated by the equations given below:

(5) $$\Delta H=E_{\mathrm{act}}-\mathrm{RT}$$

where R is the gas constant, T is the absolute temperature and E_act_ is the activation energy.

(6) $$\Delta G=-\mathrm{RT}*\ln\;\left(k_{\mathrm{cat}}\times \;h/k_{B}*T\right)$$

where h is the Planck constant (6.626 × 10^−34^ J s), and k_B_ is the Boltzmann constant (1.38 × 10^−23^ J·K^−1^).

∆S was calculated using the following equation:

(7) $$\Delta S=\left(\Delta H-\Delta G\right)/T$$

#### 2.5.4. Thermostability of Immobilized Enzyme and Kinetics of Enzyme Denaturation

Thermostability was determined by incubating the immobilized enzyme and free enzyme at 85 °C, 90 °C, and 95 °C for 5 h. Samples were taken after each hour and the residual activity was calculated at optimum temperature and pH. Enzyme activity without incubation of the enzyme at optimum conditions was taken as 100%. The data were fitted to a pseudo-first-order plot and inactivation rate constants (k_d_) were calculated at each temperature. The half-life (t_1/2_) of the enzyme at each temperature was calculated by the equation:

(8) $$t_{1/2}=\;\ln2/k_{d}\;$$

where k_d_ was calculated from the slope of the graph plotted between -ln of E/Ei (Ei is the initial enzyme activity and E is the enzyme activity at time t). The energy of activation for irreversible inactivation (Ed) of the enzyme was determined by the Arrhenius plot that was plotted as ln k_d_ versus 1000/T.

The ΔHd, ΔGd, and ΔSd of irreversible inactivation of both free and immobilized Man/Cel5B were calculated by applying Equations (5)–(7) with the modifications that in Equation (5) Ed was used instead of E_act_ and in Equation (6) k_d_ was used instead of k_cat_.

### 2.6. Reusability of Immobilized Beads

The reduction in enzyme activity of immobilized Man/Cel5B after each use was tested as described in Section 2.3. To remove any left-out substrate, the beads were removed and washed by PI buffer (pH 6.8) after each experimental run. The washed beads were mixed with fresh substrate and the residual activity was measured at optimum conditions.

### 2.7. Analysis of Chitosan and Enzyme Immobilized Chitosan Beads

The FTIR spectrum of immobilized chitosan beads was recorded from 4000 to 400 cm^−1^; using a Bruker FTIR spectrophotometer (ALPHA II Bruker) equipped with a platinum ATR sampling module and compared to the FTIR spectrum of chitosan beads without immobilization of the enzyme. Both chitosan beads and enzyme immobilized beads were lyophilized before processing for the FTIR spectrum analysis.

The crystallographic structure of chitosan and immobilized chitosan beads was determined using X-ray diffraction analysis (XRD). The XRD pattern was analyzed using Bruker D8 advance X-ray diffractometer at room temperature from 10° to 50° with a scan speed of 0.08°/s.

### 2.8. Molecular Docking Study

The homology modeling of Man/Cel5B was carried out with a SWISS-MODEL homology program [27]. The obtained model was validated by VERIFY-3D [28] and the Phi/Psi Ramachandran plot was acquired from the PROCHECK analysis tool [29]. The active sites present in the 3D model were predicted using the CASTp online server. The 3D structure of mannotriose was built in the Sybylsoftware package (Version 7.1, Tripos, Inc., St. Louis, MO, USA). Then the molecular docking of Man/Cel5B and mannotriose was performed by AutoDock software (Version 4.2, Merck & Co.GPL, Kenilworth, NJ, USA). The final structure obtained after the molecular docking studies were analyzed with the structural visualization software programs CHIMERA (Version 1.15, RBVI, UCSF, San Francisco, CA, USA) [30] and Discovery Studio Visualizer (Version 4.1, Dassault Systemes, Waltham, MA, USA) [31].

### 2.9. Statistical Analysis

All the experiments were accomplished in triplicate and the results were reported as the arithmetic mean ± standard deviation (SD). Analysis of variance (ANOVA) of data was performed with SPSS software version 22 (IBM SPSS, Armonk, NY, USA) statistical software using one-way analysis of variance, using the Tukey similarity test. *p* values < 0.05 were considered statistically significant and indicated with “*” in figures.

## 3. Results and Discussion

### 3.1. Surface Morphology of Chitosan Beads

**Figure 1:**
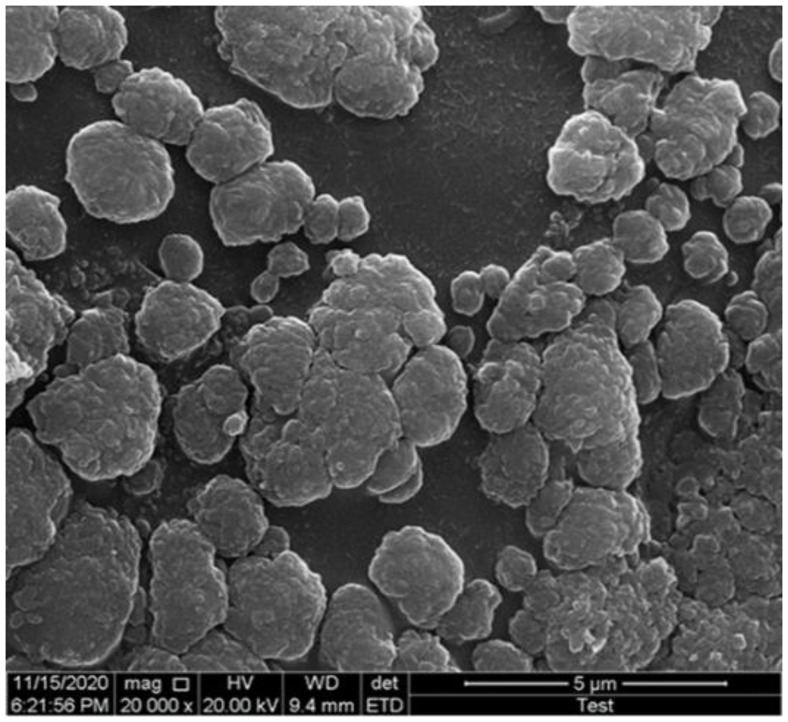
SEM micrograph of glutaraldehyde cross-linked chitosan beads.

The chitosan beads were synthesized successfully when chitosan dissolved in acetic acid was slowly dropped in NaOH solution. However, the continuous and protracted exposure may soften or disintegrate these beads. Therefore, the beads were further modified by cross-linking amine groups present in chitosan with glutaraldehyde by the establishment of the Schiff base imine bonds [32]. The beads were spherical and white in appearance. The SEM revealed that the beads were coarsely circular in shape and the surface of the beads was rough (Figure 1). The Man/Cel5B was immobilized on the prepared cross-linked beads for 5 h under shaking conditions, which resulted in 71.8% protein immobilization yield which is an important parameter to determine the percentage of enzyme that has been immobilized on or inside the solid matrix. The immobilization of thermophilic trehalose synthase on chitosan activated with glutaraldehyde by Kim et al. [33] resulted in 38% immobilization yield. Dincer et al. [34] observed 67% immobilization yield when tyrosinase was immobilized on chitosan-clay composite beads. Dhiman et al. [12] immobilized β-mannanase on sodium alginate-grafted-β-cyclodextrin, which resulted in 91.5% immobilization yield. This suggests that the enzyme leakage in our study was higher compared to study by Dhiman et al. [12], which could be improved in the future studies. The protein loading efficiency of Man/Cel5B was found to be 73.3% which was almost similar to the protein loading efficiency (78.4%) observed by Kaushal et al. [23] when catalase was immobilized on glutaraldehyde cross-linked chitosan beads.

### 3.2. Biochemical Characterization of Free and Immobilized Enzyme

#### 3.2.1. Effect of Temperature on Man/Cel5 B Immobilized Enzyme

**Figure 2:**
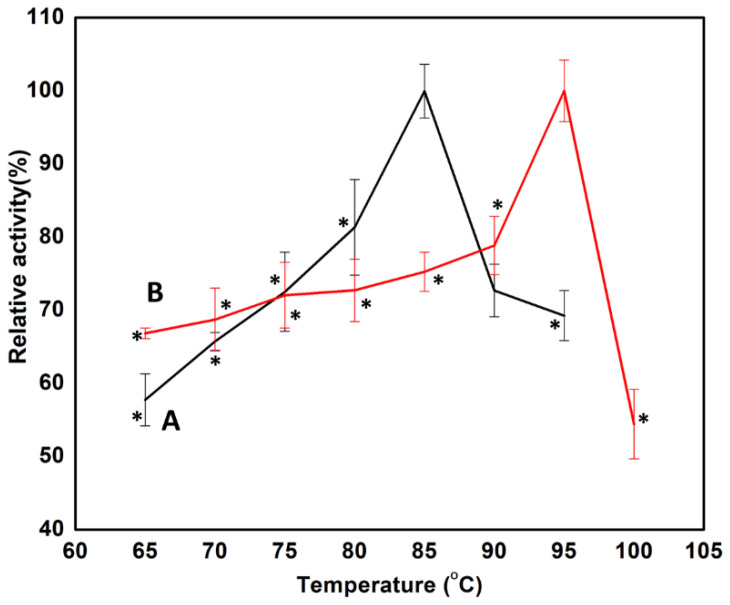
Effect of temperature on the free and immobilized Man/Cel5B activity. (A) Free Man/Cel5B. The temperature values showing significant (*p* < 0.05) difference from optimum temperature were marked with “*”. (B) Immobilized Man/Cel5B. The temperature values showing significant (*p* < 0.05) difference from optimum temperature were marked with “*”.

The temperature dependence of free and immobilized Man/Cel5B was studied between 60 and 95 °C. The optimum temperatures of free and immobilized Man/Cel5B were found to be 85 °C and 95 °C, respectively (Figure 2). The immobilized enzyme was therefore further characterized at 100 °C, where it showed a sharp decline in activity. The opti36mum temperature of the immobilized Man/Cel5B was enhanced by 10 °C which reveals that the rigidity of Man/Cel5B increased after immobilization [35]. Similarly, the optimum temperature of α-amylase from *Vigna radiata*; increased by 10 °C upon immobilization on chitosan beads [36]. The activity of the free enzyme dropped significantly when the temperature increased from 85 °C to 90 °C. Nevertheless, the activity of the immobilized enzyme continuously increased to 95 °C, which reveals that the support provides protecting effect to the enzyme at higher temperatures [37]. A similar effect of immobilization on enzymes was also observed previously [35]. The possible reason for increased optimum temperature of immobilized enzyme may be because of the synergic effect between enzyme and chitosan beads. In addition, the immobilized matrix may be able to provide a suitable micro-environment at an active center for enzyme and substrate interaction and keep its rigid confirmation and function, especially at higher temperatures [38]. However, the extent of effect varies for different support materials used and with the type of interaction established between enzyme and the solid support.

#### 3.2.2. Kinetic Paraments of Free and Immobilized Enzyme

**Figure 3:**
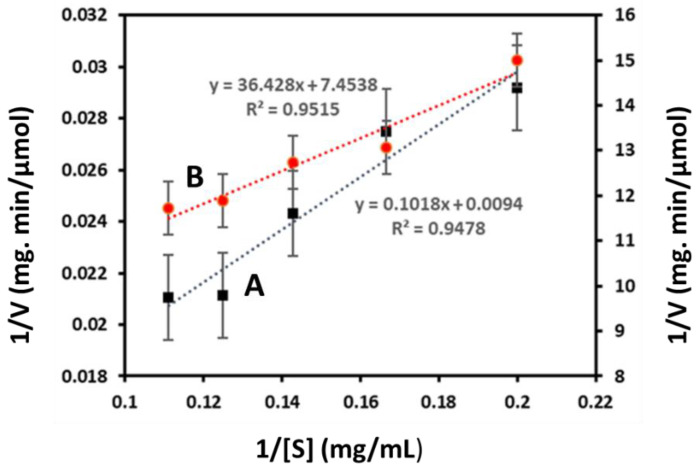
Lineweaver-Burk plots of free and immobilized Man/Cel5B. (A) Free Man/Cel5B (B) Immobilized Man/Cel5B.

The kinetic parameters of both free and immobilized enzymes were analyzed by changing the concentration of substrate (LBG) from 0.5 mg/mL to 15 mg/mL. The K_m_ values of free and immobilized Man/Cel5B were found to be 10.7 mg/mL and 4.8 mg/mL, respectively, from Lineweaver-Burk Plots (Figure 3). The result indicates that immobilized Man/Cel5B with a lower K_m_ value has significantly higher affinity for the substrate in comparison to the free enzyme. Possibly, the Man/Cel5B expanded on the surface of chitosan beads with a better orientation which resulted in more available active sites for substrate binding that eventually causes higher affinity and lower K_m_ value. The decrease in the Km value after immobilization is a desirable property and many authors reported significant decrease in K_m_ after immobilization of the enzymes. The decrease in K_m_ value could be due to increased availability of substrate molecule to the active site of the enzyme molecule [39]. When the enzyme was immobilized on silica-coated modified magnetite nanoparticles, the K_m_ value of α-Amylase decreased from 6.3 mM to 4.8 mM [39]. Similarly, the K_m_ value of α-Amylase decreased after immobilization on bioactive phospho-silicate glass [40].

Furthermore, the V_max_ of free and chitosan immobilized Man/Cel5B was found to be 106 µmol (min mg protein)^−1^ and 2.56 µmol (min mg protein)^−1^, respectively. The decrease in the V_max_ was also observed by Çetinus and Oztop [41] when catalase was immobilized on chitosan beads. The V_max_ of beta-mannanase immobilized on Na-alginate-βCD was also lower than the free enzyme [12]. Although higher Vmax after immobilization is a desirable property, the reason for V_max_ being lower than the free enzyme system could be because of the conformational changes in the enzyme after immobilization on the support material. Another reason may be seepage or congestion of the enzyme during the adsorption process of adsorbate-adsorbent interaction [12]. These parameters should be better optimized in future studies.

#### 3.2.3. Thermodynamics of LBG Hydrolysis

**Figure 4:**
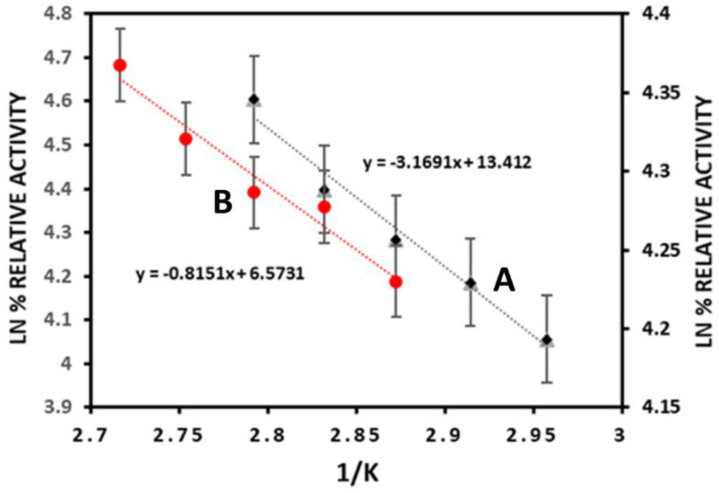
Arrhenius plots for free and immobilized Man/Cel5B. (A) Free Man/Cel5B (B) Immobilized Man/Cel5B.

The calculated activation energy (E_act_) of free and immobilized enzymes from Arrhenius plots (Figure 4) depicted a reduction from 26.34 J/mol to 6.64 J/mol (Table 1). Similarly, the E_act_ of β-mannanase decreased from 28.19 kJmol^−1^ to 23.52 kJmol^−1^ after immobilization on sodium-alginate-grafted-β-cyclodextran [12]. A similar observation was also reported when mannanase from *P. chrysogenum* was immobilized by entrapment on calcium alginate beads [42]. The decrease in E_act_ indicates that immobilized enzyme requires less energy to overcome the barrier for conversion of substrate into product. This conceivably means that the immobilization process saves energy which makes the immobilized enzyme more valuable economically: a vital advantage for industrial use of the enzyme [12]. The decrease in the activation energy also indicated a lower sensitivity to the temperature and higher affinity for the active site of the immobilized enzyme [43].

The thermodynamic parameters ΔH, ΔG, and ΔS were also calculated and the results are shown in Table 1. The ΔH for free and immobilized enzymes were found to be 23.37 KJ/mol and 3.72 KJ/mol, respectively. The decrease in enthalpy value demonstrates that the formation of the enzyme-substrate complex was more efficient for the immobilized Man/Cel5B compared to the free enzyme [44]. The ΔG for the immobilized enzymes was found to be 85.26 KJ/mol which was slightly higher than the ΔG observed for the free enzyme, which is 72.68 KJ/mol. The increase in ΔG indicates that conversion of substrate-enzyme transition complex was more spontaneous for the free enzyme compared to the immobilized enzyme. The ΔS for immobilized and free enzymes were found to be −221.33 J/mol/K and −137.74 J/mol/K, correspondingly. The lower value of ΔS indicates a lesser disorder for the transition complex of the immobilized enzyme system [45].

#### 3.2.4. Thermostability of Free and Immobilized Man/Cel5B

**Figure 5:**
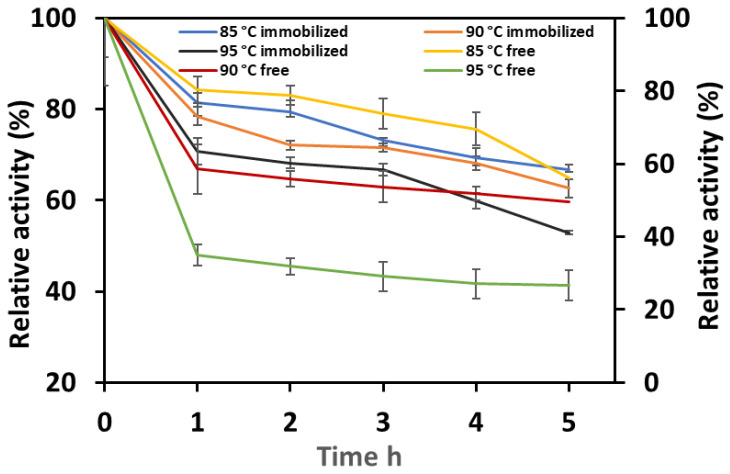
Thermostability of free and immobilized Man/Cel5B. The enzymes were incubated at 80, 85 and 90 °C for 5 h in the absence of substrate, and the residual activity was examined at optimal conditions.

The immobilized and free Man/Cel5B were incubated at 85 °C, 90 °C, and 95 °C. The results (Figure 5) clearly show that thermostability increased after immobilization of Man/Cel5B on chitosan beads. The enzyme was able to retain >65% of its initial activity after 5 h of incubation at 85 °C compared to 55% of the free enzyme. At higher temperatures, the free enzyme lost activity more rapidly compared to the immobilized enzyme. These results suggest that the free enzyme was exposed to conformational changes as the temperature of reaction system increased, which resulted in rapid denaturation of enzyme and consequently loss of activity. However, for immobilized enzyme the interaction between support and enzyme provides more stable conformation which protects the immobilized enzyme at higher temperatures [46]. The immobilized enzyme retained more than 60% and 50% of activity after 5 h of incubation at 90 °C and 95 °C, respectively. However, the free enzyme could only retain 49% and 26% of initial enzyme activity after 5 h at 90 °C and 95 °C, respectively. The extent of thermal inactivation was determined using Pseudo-first-order plots, and thermal inactivation constants (k_d_) were calculated for both types of enzyme systems. The k_d_ values (Table 2) were found to be 0.074/h, 0.079/h and 0.106/h for immobilized; and 0.097/h, 0.11/h, and 0.22/h at 85, 90 and 95 °C respectively. Similarly, the k_d_ values of lipase immobilized on sol-gel decreased from 0.02/h to 0.01/h [47]. The decrease in k_d_ indicates higher stability of enzyme at elevated temperatures which suggests that immobilization protected the tertiary structure of enzyme by preventing denaturation [46]. The decimal reduction time (D-value) of immobilized Man/Cel5B increased to 1864, 1742 and 1403 from 1424, 1244, 611 at 85, 90 and 95 °C, respectively. This may be because of the formation of multiple covalent bonds between enzyme and crosslinked chitosan beads, which result in a reduction of conformational flexibility and thermal vibration; accordingly protein unfolding and denaturation were prevented [48]. Moreover, the half-life (t_1/2_) of the enzyme considerably increased after immobilization, which was found to be 9 h for the immobilized enzyme compared to only 7 h for the free enzyme at 85 °C (Table 2). The half-life of both free and immobilized enzymes decreased at higher temperatures; however, the half-life of the immobilized enzyme was found to be higher than the free enzyme also at higher temperatures. The increased half-life of the immobilized enzyme complex indicates high thermal stability of the immobilized Man/Cel5B. Altun et al., also observed increase in the thermostability of pepsin after immobilization on chitosan beads [43]. The structural rigidity and heat resistance of the enzyme can increase to a great extent after attachment to a suitable support [49]. This results in the decreased sturdiness of free enzymes compared to immobilized enzyme at same temperatures [50].

#### 3.2.5. Thermodynamics of Enzyme Stability after Immobilization

**Figure 6:**
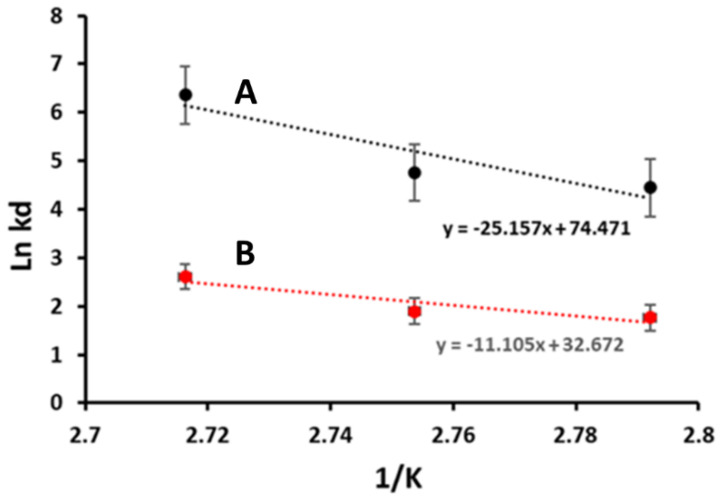
Arrhenius plots to calculate activation energy “Ed” for irreversible thermal inactivation/denaturation of free and immobilized Man/Cel5B. (A) Free Man/Cel5B. (B) Immobilized Man/Cel5B.

The Ed values for the free and immobilized Man/Cel5B were determined using Arrhenius plots (Figure 6) which were found to be 92 KJmol^−1^ and 209 KJmol^−1^, respectively. The increase in E_d_ indicates that immobilized Man/Cel5B is more resistant to heat denaturation, therefore in need of excess energy as compared to the free enzyme [44], which is a desirable characteristic for industrial use of immobilized Man/Cel5B. The E_d_ is also related to another important thermodynamic parameter known as enthalpy of activation for thermal denaturation ΔH_d_. The value of ΔH_d_ for immobilized Man/Cel5B was higher at all temperatures than the free Man/Cel5B (Table 2). The higher values of E_d_ and ΔH_d_ indicate high thermal stability of the immobilized Man/Cel5B however, it is also important to note the values of ΔG_d_ and ΔS_d_. As indicated in Table 2, the ΔG_d_ values of the immobilized enzyme were higher than the free enzyme at all temperatures. The ΔG_d_ values depict how favorable is the process of inactivation at a particular temperature. Lower ΔG_d_ values of free enzyme indicate the process of thermal denaturation for the free enzyme is more spontaneous compared to immobilized enzyme complex. These results also suggest that interaction between support and enzyme provides more stable conformation which prevents the tertiary structure of enzyme from denaturation. The ΔG_d_ values also depict higher thermal stability of immobilized enzyme than free Man/Cel5B which increases its value for industrial use. Furthermore, the lower ΔS_d_ indicates a decrease in the degree of disorder for the immobilized enzyme system [51].

#### 3.2.6. Effect of pH on Free and Immobilized Man/Cel5B

**Figure 7:**
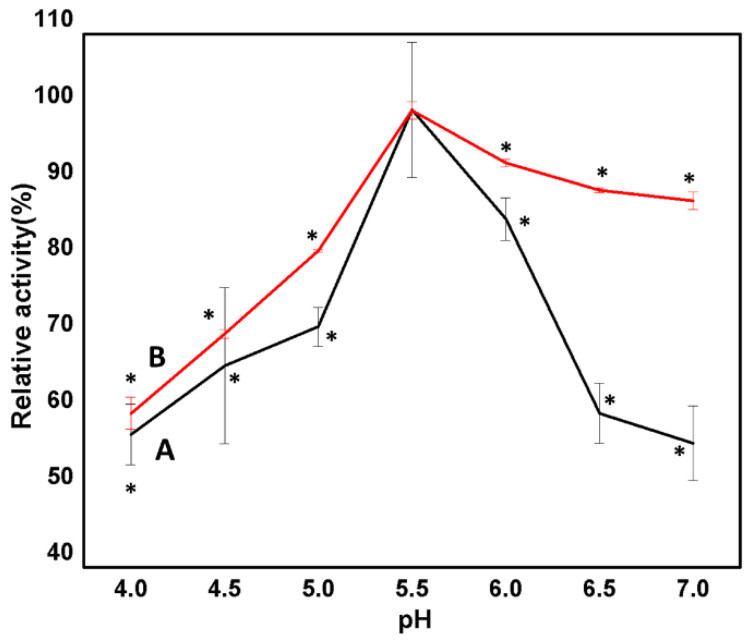
Effect of pH on the free and immobilized Man/Cel5B activity. (A) Free Man/Cel5B. The pH values showing significant (*p* < 0.05) difference from optimum pH were marked with “*”. (B) Immobilized Man/Cel5B. The pH values showing significant (*p* < 0.05) difference from optimum pH were marked with “*”.

The process of immobilization affects the ionization state, dissociation constant, and conformation of the enzyme. As a result, the relationship between pH, stability, and catalytic activity of the enzyme changes. To determine the effect of immobilization on the chemical characterization of the enzyme, the optimum pH of the immobilized enzyme was compared with the free Man/Cel5B (Figure 7). The effect of pH was determined in the range of pH 4–7 using PI buffer at optimum temperature (85 °C and 95 °C for free and immobilized enzyme, respectively). The optimum pH for both forms of Man/Cel5B enzyme was found to be pH 5.5. The chitosan beads may become disintegrated in acidic medium after long experimental exposures. In a study carried out by Worthen et al. [52] chitosan beads remained unchanged after the first seven hours at pH 5; however, the beads started to slowly shrink after 8 h and were almost disintegrated after 16 h of exposure to pH 5. However, in the current study, the immobilized enzyme showed the optimum activity at pH 5.5, which should be examined in the future studies. Additionally, it may be possible that crosslinking enhances the pH stability of chitosan beads, which was also observed by Worthen et al. [52], when chitosan beads were crosslinked by Tripolyphosphate. However, the effect of glutaraldehyde must be explored in future studies. The immobilized enzyme depicted enhanced activity in the pH range of 5.5–7. This enhanced activity of immobilized enzyme toward neutral pH may be as a result of the covalent binding of the enzyme with chitosan and change in the immediate microenvironment of the enzyme [8]. Our observations were in congruence with the findings of Mohapatra et al., who demonstrated no change in the optimum pH of β-mannanase from *Streptomyces* spp. Alg-S25 after immobilization on chitosan nanoparticles [53]. Similarly, the optimum pH of bovine liver catalase upon immobilization on chitosan beads was found be to be same as its native form [8]. However, there are many reports where the optimum pH of the enzyme shifted after immobilization on the chitosan beads. For example, the optimum pH of β-mannanase from *Penicillium occitanis* decreased to pH 3 from pH 4 when the enzyme was immobilized on chitosan bentonite beads [54]. This shows that different enzymes do behave differently to the change in pH of the immediate environment when immobilized on chitosan beads.

### 3.3. Reusability of Immobilized Man/Cel5B

**Figure 8:**
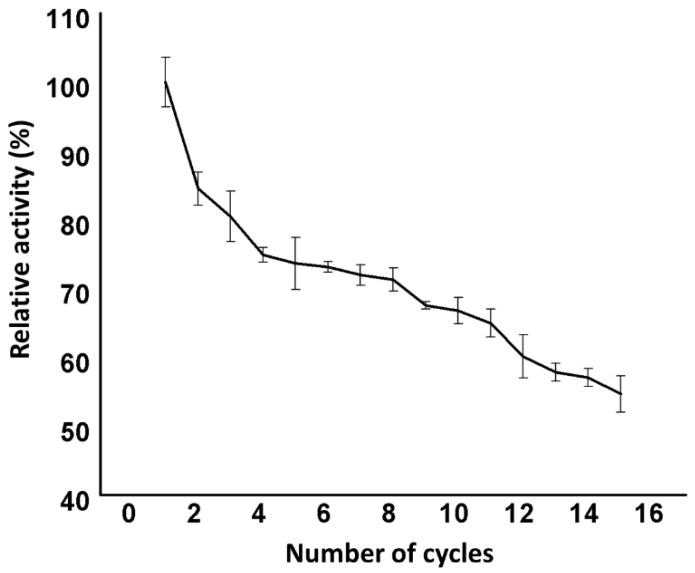
Reusability of the immobilized Man/Cel5B.

The reusability of immobilized enzymes is an important parameter from the economical point of view, as well as for the industrial use of enzymes. The reusability of immobilized Man/Cel5B was evaluated 15 times at the optimum temperature and pH (Figure 8). The immobilized enzyme showed excellent reusability as it retained almost 85% of the initial activity after the first cycle. The enzyme started losing its activity gradually after each use, and retained almost 54% of the initial activity after 15 uses. These results indicate that the immobilized enzyme could be used for industrial applications. The sodium-alginate-β-cyclodextrin immobilized mannanase retained more than 70% of activity after 15 repeated cycles [12]. Laccase immobilized on SiO_2_ nanoparticles retained 94% of its initial activity after 5 cycles of repeated use, and 83% activity after 10 cycles [55]. The decrease in the enzyme activity may be due to the denaturation of bonds, as well as because of the enzyme leakage [28].

### 3.4. Fourier-Transform Infrared Spectroscopy (FTIR) Spectrum of Chitosan and Chitosan Immobilized Beads

**Figure 9:**
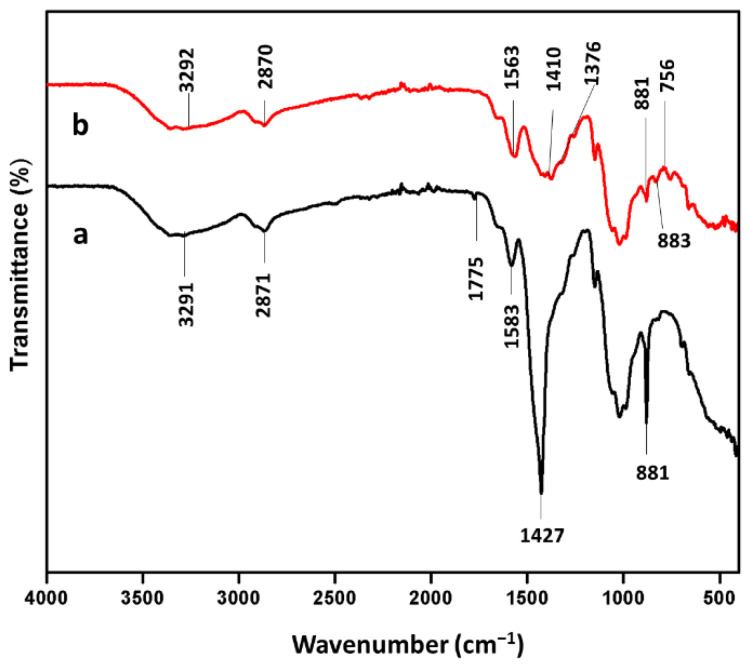
FTIR spectra of chitosan beads and Man/Cel5B immobilized chitosan beads. (a) Chitosan beads. (b) Immobilized chitosan beads.

FTIR analysis was performed to illustrate the functional groups present in the glutaraldehyde reinforced chitosan beads and Man/Cel5B immobilized beads (Figure 9). The main peaks for glutaraldehyde reinforced chitosan beads can be assigned as follows: 1022 cm^−1^. 71 (C-OH stretching vibration), 1149 cm^−1^ (monosaccharide structure present in chitosan polymer), 1427 cm^−1^ (C-N stretching vibration), 1583 cm^−1^ (N-H bond), and 3293 cm^−1^ (N-H and O-H stretching vibration) [56]. In addition to this, the band at 1775 cm^−1^ maybe because of the CHO group present in glutaraldehyde [55]. Nevertheless, some major changes were observed in the FTIR spectrum of Man/Cel5B immobilized chitosan beads when compared to the FTIR spectrum of glutaraldehyde reinforced chitosan beads. The FTIR spectrum of Man/Cel5B immobilized chitosan beads reveals that the N-H and O-H stretching vibration at 3293 cm^−1^ shifts to 3292 cm^−1^. The appearance of band at 1410.10 cm^−1^ could be attributed to CH stretching vibration of β-mannanase [55]. Moreover, the band at 1563.05 cm^−1^ could be attributed to the N-H bending vibration of the amide II. These results also agree with the immobilization of β-galactosidase on chitosan, and cellulase on chitosan magnetic nanoparticles [57].

### 3.5. X-ray Powder Diffraction (XRD) of Chitosan and Chitosan Immobilized Beads

**Figure 10:**
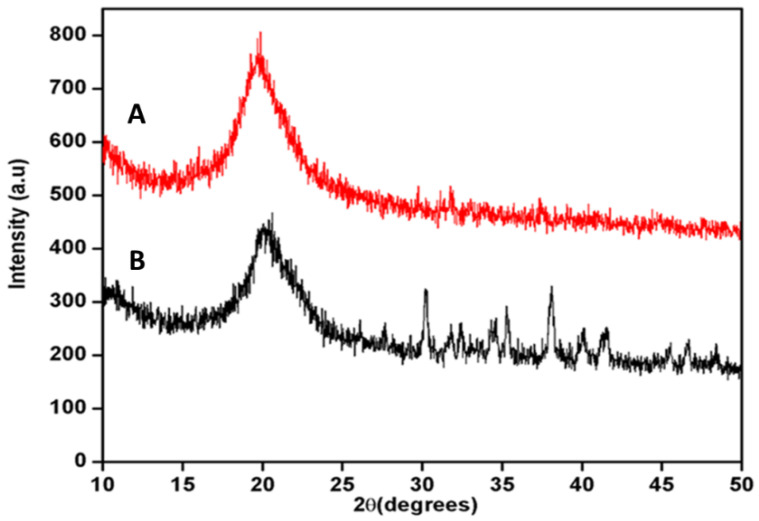
XRD spectra of chitosan beads and Man/Cel5B immobilized beads. (A) Chitosan beads. (B) Man/Cel5B immobilized chitosan beads.

The change in the crystal structure of chitosan beads before and after immobilization of Man/Cel5B was investigated using XRD. The XRD is a useful tool to study crystal lattice arrangements and provides valuable information about the degree of crystallinity in the sample. The XRD patterns of chitosan beads and Man/Cel5B-immobilized chitosan beads are shown in Figure 10. The XRD pattern of chitosan beads depicted a broad diffraction peak at 2θ = 20°, which is the characteristic peak for crystal chitosan. The additional peaks were observed at 2θ = 30°, 35°, and 38° of Man/Cel5B immobilized chitosan beads. The appearance of new peaks confirms the strong binding of chitosan beads with the Man/Cel5B beads.

### 3.6. Molecular Docking Analysis

**Figure 11:**
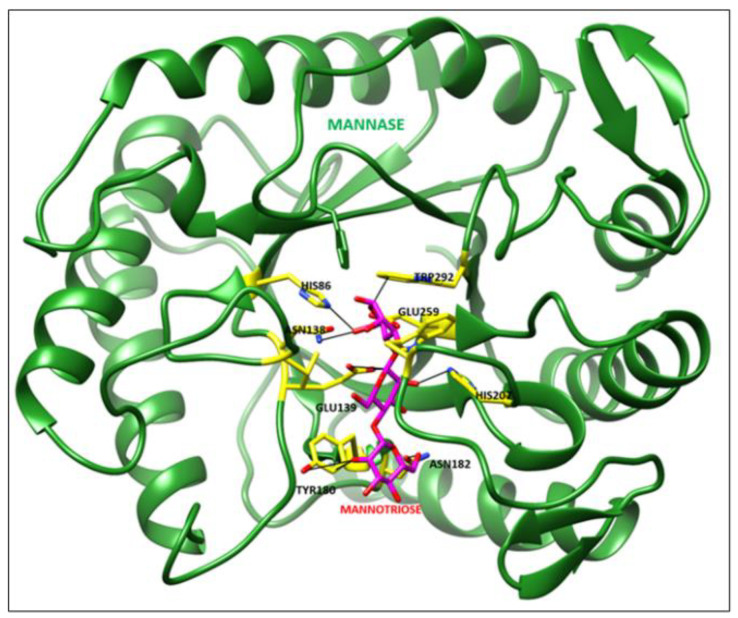
Docked complex of Mannanase (Green) with Mannotriose (Magenta).

**Figure 12:**
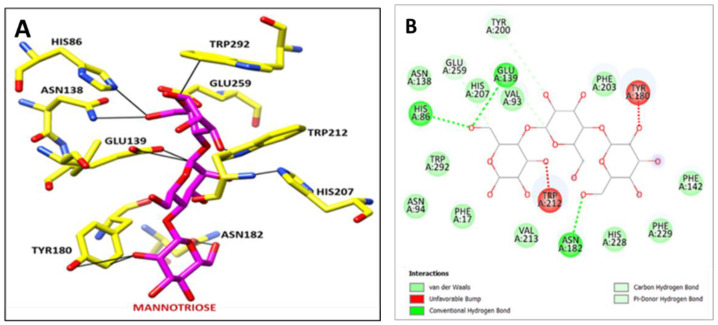
(**A**) Bonding interaction of active site residues of Mannanase (yellow) with Mannotriose (Magenta). (**B**) 2D-interactions between active site residues of mannanase with mannotriose.

The Psi Ramachandra plot obtained from PROCHECK analysis displayed that 88.20% amino acid residues of Man/Cel5B reside in the most favored region, and 11.80% of amino acid residues in additional allowed regions and no amino acid residue were found in generously allowed or disallowed regions (Figure S1). Further Verify-3D analysis was carried out to check the quality of the predicted mannanase structure. The Verify-3D depicted that the average 3D-ID score of 97.54% of the amino acid residues predicted model of Man/Cel5B is >0.2 which strongly indicates that the predicted structure is of adequate quality. The CASTp analysis showed that the amino acid residues such as Phe17, His86, Val93, Asn94, Lys95, Glu139, Phe142, Gly179, Tyr180, Asn182, Tyr200, Ile201, Phe203, His207, Trp212, Val213, His229, Glu259, Trp292, and Phe298 might play an important role at catalytic site (Figure S2). The molecular docking analysis revealed that the Man/Cel5B structure has strong hydrogen-bonding interactions with the mannotriose ligand (Figure 11). The docked complex showed that there are strong hydrogen-bonding interactions between Man/Cel5B active site residue His86 and mannotriose (BMA 1.C O6) by interatomic distance of 2.6 Å (Figure 12A,B; Table 3). Similarly, Glu 139 (A OE1) interacts with mannotriose (BMA 2.C C1:) by interatomic distance of 2.7 Å. Additionally, the docked complex showed strong hydrogen-bonding of active site residues such as Tyr180, Asn182, His207, Trp212, Glu259, and Trp292 with mannotriose. Hence form this docking of β-mannanase with mannotriose showed very strong hydrogen-bonding interactions with mannotriose ligand.

## 4. Conclusions

The purified Man/Cel5B was successfully immobilized on glutaraldehyde cross-linked chitosan beads with an immobilization yield of 71.8%. The immobilized enzyme depicted its optimal activity at pH 5.5 and 95 °C compared to pH 5.5 and 85 °C for the free enzyme. The immobilized enzyme revealed better thermodynamic properties compared to the soluble enzyme which is an essential feature for possible industrial use of β-mannanase. The substrate affinity for LBG of the immobilized enzyme complex was higher than the soluble enzyme. All these features make chitosan immobilized Man/Cel5B an promising candidate for many biotechnological applications.

## Figures and Tables

**Table 1:** Thermodynamic parameters of immobilized and free enzyme for LBG degradation.

Thermodynamic Parameter	Immobilized Enzyme	Free Enzyme
E_act_ (KJmol^−1^)	6.78 ± 0.88	26.35 ± 3.33
ΔH (KJmol^−1^)	3.72 ± 0.26	23.37 ± 2.68
ΔG (KJmol^−1^)	85.17 ± 3.26	72.68 ± 4.21
ΔS (J·mol^−1^·K^−1^)	−221.33 ± 5.13	−137.74 ± 4.49

**Table 2:** Thermodynamic parameters for irreversible inactivation of free and immobilized Man/Cel5B.

Parameter	Immobilized Enzyme			Free Enzyme		
Temperature (°C)	85	90	95	85	90	95
K_d_ (min^−1^)	4.45 ± 0.03	4.75 ± 0.99	6.63 ± 1.23	5.82 ± 0.42	6.66 ± 0.24	13.5 ± 0.44
t_1/2_ (min^−1^)	561 ± 2.12	524 ± 3.30	392 ± 3.63	428 ± 1.35	374 ± 2.63	184 ± 3.33
D-value	1864 ± 8.53	1742 ± 4.12	1303 ± 1.67	1424 ± 3.33	1244 ± 6.55	611 ± 5.45
ΔH_d_ (KJmol^−1^)	206.24 ± 2.04	206.20 ± 2.85	206.16 ± 1.35	89.41 ± 2.45	89.37 ± 1.84	89.33 ± 2.63
ΔG_d_ (KJmol^−1^)	82.80 ± 1.48	84.00 ± 2.43	85.20 ± 1.67	72.71 ± 1.64	73.77 ± 2.50	74.82 ± 8.46
ΔS_d_ (J·mol^−1^·K^−1^)	−230.63 ± 1.90	−230.75 ± 4.44	−230.87 ± 3.24	−202.76 ± 3.44	−202.89 ± 1.53	−203.01 ± 2.43

**Table 3:** Hydrogen-bonding interactions between mannanase with mannotriose.

Sr. No.	Interaction between Amino Acid Residues of Mannanase with Mannotriose	Distance in Å
1	BMA 1.C O5 ------ TRP 292.A CZ2:	3.302
2	HIS 86.A NE2 ------ BMA 1.C O6:	2.645
3	ASN 138.A ND2 ------ BMA 1.C O6:	3.473
4	BMA 1.C C6 ------ GLU 259.A OE1:	3.319
5	TRP 212.A NE1 ------ BMA 1.C O3:	1.243
6	BMA 2.C O2 ------ HIS 207.A NE2:	3.039
7	GLU 139.A OE1 ------ BMA 2.C C1:	2.769
8	TYR 180.A CE2 ------ BMA 3.C O2:	1.721
9	ASN 182.A OD1 ------ BMA 3.C O6:	2.344

## Data Availability

Not applicable.

## Supplementary Materials

The following supporting information can be downloaded at: https://www.mdpi.com/article/10.3390/biom12070999/s1, Figure S1: Ramachandran Plot of Man/Cel5B structure; Figure S2: Structural validation using Verify-3D 3D/1D profile; Figure S3: Active site prediction for Man/Cel5B structure using CASTp server.
