# Supplementary material for: Modifying Thermostability and Reusability of Hyperthermophilic Mannanase by Immobilization on Glutaraldehyde Cross-Linked Chitosan Beads
Source: Biomolecules. 2022 Jul 18;12(7):999. doi: 10.3390/biom12070999 (PMC9312517; doi:10.3390/biom12070999)
Supplement: Supplementary file 1 [file biomolecules-12-00999-s001.zip › biomolecules-1806977-supplementary.pdf]

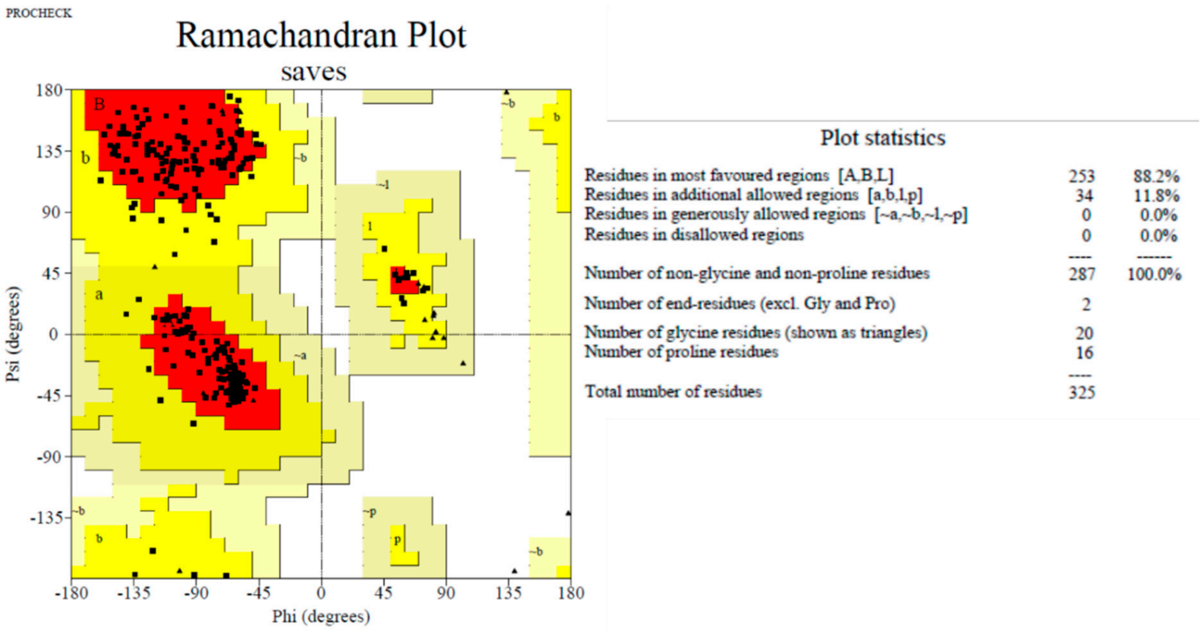

Figure S1: Ramachandran Plot of Man/Cel5B structure.

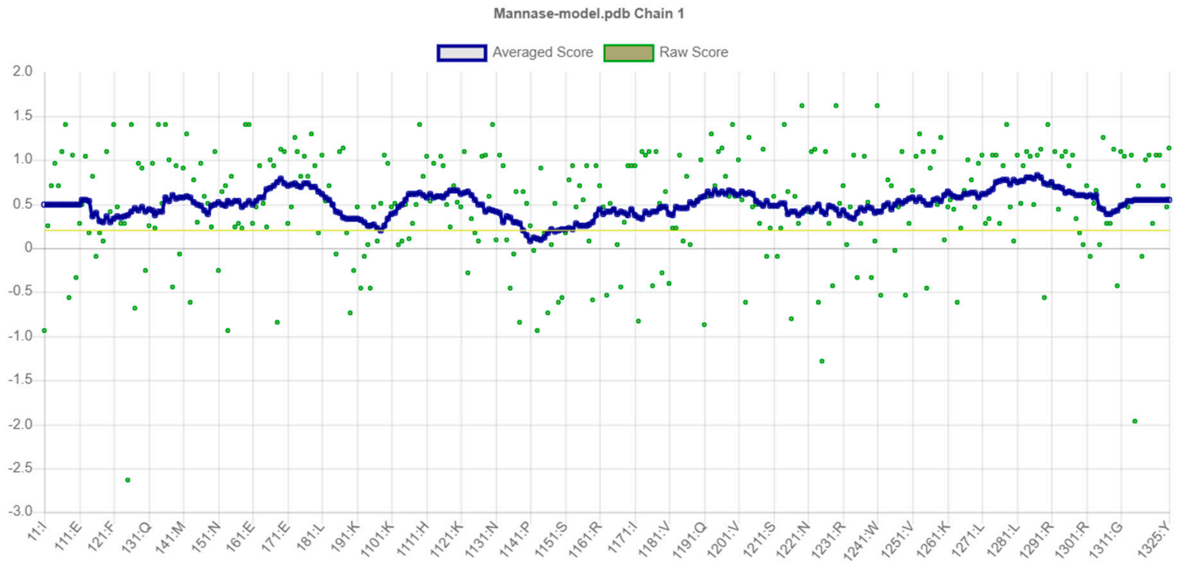

Figure S2: Structural validation using Verify-3D 3D/1D profile.

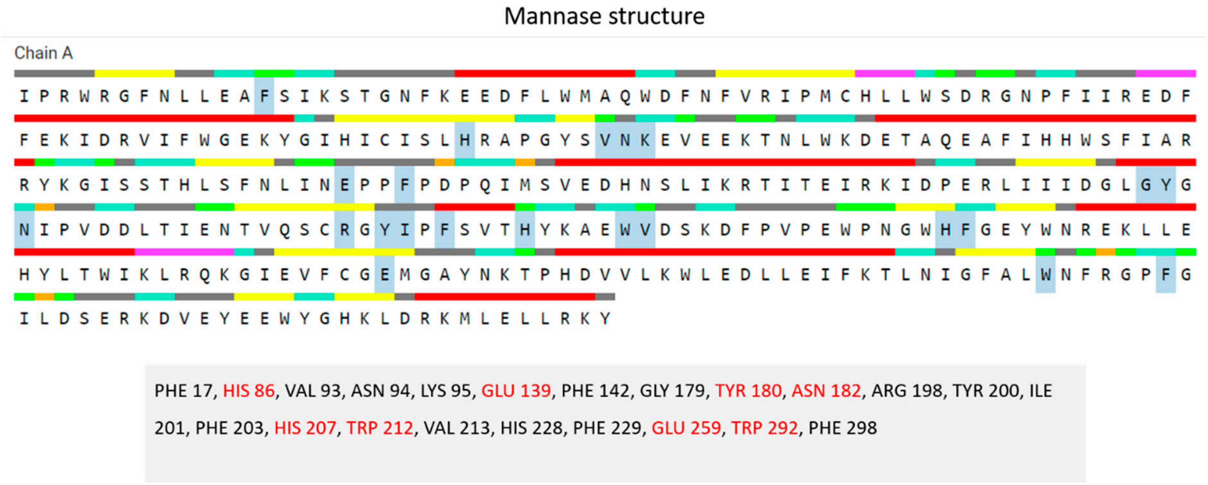

Figure S3: Active site prediction for Man/Cel5B structure using CASTp server.
